# De Novo Partial 13q22-q34 Trisomy with Typical Neurological and Immunological Findings: A Case Report with New Genetic Insights

**DOI:** 10.3390/brainsci11010021

**Published:** 2020-12-26

**Authors:** Claudia Brogna, Valentina Milano, Barbara Brogna, Lara Cristiano, Giuseppe Rovere, Roberto De Sanctis, Domenico M. Romeo, Eugenio Mercuri, Giuseppe Zampino

**Affiliations:** 1 Pediatric Neurology Unit, Fondazione Policlinico Universitario “A. Gemelli”, IRCSS, Università Cattolica del Sacro Cuore, 00168 Rome, Italy; laracristiano2811@gmail.com (L.C.); robertodesantis@yahoo.it (R.D.S.); domenicomarco.romeo@policlinicogemelli.it (D.M.R.); eugeniomercuri@unicatt.it (E.M.); 2 Neuropsychiatric Unit, ASL Avellino, 83100 Avellino, Italy; 3 Genetic Unit, Fondazione Policlinico Universitario A. Gemelli, IRCSS, Università Cattolica del Sacro Cuore, 00168 Rome, Italy; milvale@gmail.com; 4 Pediatric Unit, Sapienza University, 00100 Rome, Italy; 5 Radiology Unit, S.G. Moscati Hospital, 83100 Avellino, Italy; brognabarbara1@gmail.com; 6 Orthopedic Unit, Fondazione Policlinico Universitario “A. Gemelli”, IRCSS, Università Cattolica del Sacro Cuore, 00168 Rome, Italy; rovere292@hotmail.com; 7 Pediatric Unit, Fondazione Policlinico Universitario “A. Gemelli”, IRCSS, Università Cattolica del Sacro Cuore, 00168 Rome, Italy; Giuseppe.Zampino@unicatt.it

**Keywords:** partial trisomy 13q, hemiparesis, epilepsy, ischemic and haemorrhagic cerebral lesions

## Abstract

The partial trisomy 13q encompasses an extensive variability of phenotypic and radiological findings including leukoencephalopathy and brain malformations such as holoprosencephaly, callosal dysgenesis, hippocampal hypoplasia, olfactory hypoplasia, and vermian hypoplasia. We report for the first time a case of a 23-year-old patient affected by de novo partial 13q22.1q34 trisomy (41.7 Mb, 72,365,975-114,077,122x3) presenting with hemiparesis related to both ischemic and haemorrhagic cerebral lesions compatible with cerebral vasculitis due to a possible combination of genetic and immunological interaction.

## 1. Introduction

Partial 13q trisomy is an uncommon genetic condition, characterized by an extensive variability of phenotypic expression. The few cases reported in the literature are related to an unbalanced translocation, while isolated de novo duplications of the distal 13 q are very rarely stated [1,2,3]. Major phenotypic features include psychomotor delay, intellectual disability and specific facial morphological anomalies as well as long philtrum, frontal bossing, stubby nose, haemangioma mainly associated to the involvement of the q22 segment and, less frequently, microcephaly, hypertelorism and hexadactyly [3,4]. Some radiological findings associated to partial 13 trisomy are leukoencephalopathy, bilateral perisylvian and rolandic cortical dysplasia, brain malformations including holoprosencephaly, callosal dysgenesis, hippocampal hypoplasia, olfactory hypoplasia, vermian hypoplasia [4]. Similarly, epileptic seizures often occurred, related to the presence of structural cerebral lesions or to the dysfunctional neuronal neurotransmition regulation typical of the chomosomopathies. However, so far, only few reports described radiological findings typical of ischemic cerebral lesions related to partial 13q trisomy while the presence of stroke related to abnormal coagulation pattern is not yet well investigated [4]. We report the clinical case of a 23-year-old patient affected by de novo partial 13q22-q34 trisomy (41.7 Mb) presenting with hemiparesis related to both ischemic and haemorrhagic cerebral lesions compatible with cerebral vasculitis associated with immunological findings.

### Case Presentation

A 23-year-old male was born at term age after a normal pregnancy from non-consanguineous parents. Family history was positive for developmental disorders including autism spectrum disorder and intellectual disability (two children of a cousin of the 1st grade mother). The delivery occurred with an emergency caesarean section due to the presence of umbilical cord around the neck of the baby and cardiotocometric trace abnormalities. At birth, a mild distress occurred, associated to a mild hypotonia. Postaxial polydactyly of the hands was also found. Mild motor development delay was reported during infancy (independent walking reached at the age of 2 years) while no speech disturbance was reported.

The first generalized epileptic seizures occurred when he was 3 years old. Seizures were initially treated with valproate acid and levetiracetam, thereafter replaced by carbamazepine, zonesamide and phenobarbital due to the evolution into drug-resistant epilepsy. Magnetic resonance imaging performed at the age of 3 years showed a mild leukoencephalopathy. Karyotype analysis confirmed the presence of an inverted and duplicated partial trisomy involving the 13q22.1 qter region.

**Figure 1:**
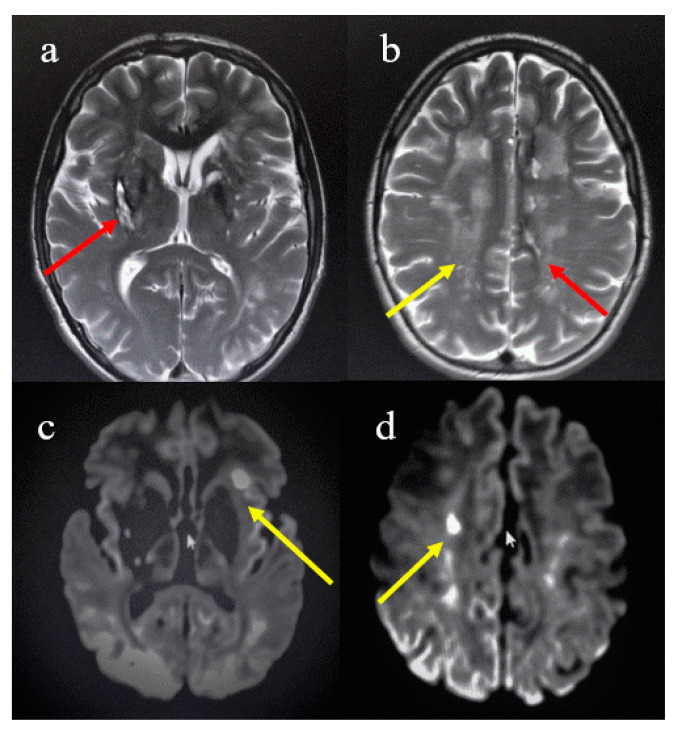
First brain MRI (3 months later the onset of hemiparesis). In the image “**a**”, T2 weighted sequence shows an hypertense area with hypointense ring compatible with the chronic phase evolution of the previous hemorrhage in the right basal ganglia (red arrow). In the image “**b**”, T2 weighted sequence shows white matter alterations with leukoencephalopathy (yellow arrow) and hypointense signal in some left cerebral sulci at brain convexity compatible with hemosiderin foci (red arrow); images “**c**” and “**d**” report DWI sequences with restricted diffusion lacunar areas compatible with subacute ischemic lesions in the left frontal-orbital subcortical area (yellow arrow in the image “**c**”) and in the correspondence of the corona radiate (yellow arrow in the image “**d**”).

Several years later, at the age of 23, he abruptly presented with drop attack, dysarthria, and left hemiparesis. A first cerebral cranial Tomography (CT) requested in emergency described a hemorrhagic area within the right basal ganglia associated with different small hyperdense lesions in the periventricular area, while an echocardiogram exam revealed a mitral prolapse associated with a mild left ventricle hypertrophy. Therefore, anti-hypertensive therapy was started. A brain magnetic resonance imaging (MRI) scan was performed 3 months later with a Philips device at 1.5 Tesla-field and T1, T2, and Fluid Attenuation Inversion Recovery weighted (FLAIR) images were obtained. The MRI showed a chronic evolution of the previous hemorrhagic area within the right basal ganglia and also the presence of hemosiderin foci in some cerebral sulci in the left cerebral convexity (Figure 1a,b). Subcortical leukoencephalopathy was also visible with lacunar subacute ischemic lesions in the left frontal-orbital subcortical area and bilaterally in the white matter at the level of the corona radiata (Figure 1a–c). The findings reporting both ischemic and hemorrhagic areas were compatible with cerebral vasculitis. An autoimmunity assessment and a thrombophilia screen revealed normal results except for elevated levels of hyperhomocysteinaemia (31.1 µM/L) (normal range between 5 and 15 µM/L) in presence of heterozygotic MTHFR C677T polymorphism and normal level of folate and vitamin B12. Autoimmune assessment and antiphospholipid antibodies (aPL) including lupus anticoagulant (LAC), anti-cardiolipin antibodies (IgG and IgM) and anti-β2-glycoprotein I antibodies (IgG and IgM) were also investigated as part of thrombophilia assessment and were reported as normal. Epiaortic ultrasonography was negative for stenosis. Six months later, neurological and orthopedic examination confirmed the presence of left hemiparesis (upper limb involved more than lower limb), dysarthria, facial morphological anomalies, and moderate intellectual disability. The autoimmune and thrombophilia assessment including aPL revealed normal results except for LAC positivity detected by diluted Russell viper venom time (dRVVT) assay (coagulation time of dRVVT 39.4 s confirmed by dRVVT ratio value 1.26) and hyperhomocysteinaemia (20.6 µM/L). Blood test showed no relevant abnormalities in hematology or biochemistry including normal renal and liver function. Total cholesterol and LDL cholesterol were also normal as well as factor VIII. Considering the presence of different clinical signs including intellectual disability, both ischemic and hemorrhagic cerebral lesions, mitral prolapse and a mild left ventricle hypertrophy, further analysis were performed including an array-comparative genomic hybridization (array-CGH) using the SurePrint G3 Human CGH 4x180K Microarray kit (Agilent Te1chnologies, Santa Clara, CA, USA) on genomic DNA from the blood. The results confirmed the presence of de-novo 13q22-q34 duplication (41.7 Mb, 72,365,975-114,077,122x3) including *GPC5* (OMIM 602446) and *GPC6* (OMIM 604404), *COL4A1* (OMIM 120130) and *COL4A2* (OMIM 120090), *PROZ* (OMIM 176895), *GAS6* (OMIM 600441), *GPR180* (OMIM 607787), *STK24* (OMIM 604984), *TNFSF13B* (OMIM 603969) and *NALCL* (OMIM 611549) genes.

**Figure 2:**
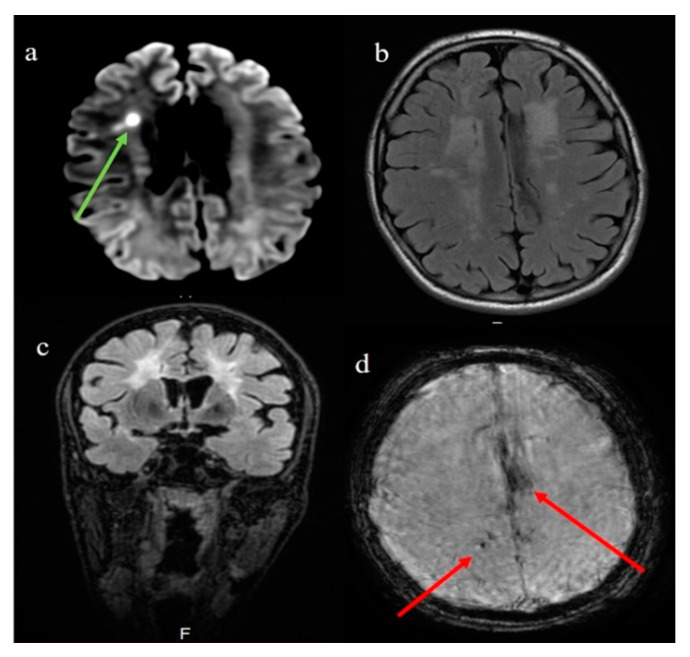
Second brain MRI (1 year later). In the image “**a**” the DWI sequence shows a restricted diffusion in the right periventricular area compatible with subacute ischemic lesion (green arrow); Flair sequences in images “**b**” and “**c**” show a mildly more extensive subcortical leukoencephalopathy; in the image “**d**” SWI sequence highlights the left superficial hemosiderosis in the brain sulci at convexity (red arrow) with right microbleeds (red arrow).

One year later, an MRI follow-up scan was performed showing a new little ischemic lesion in the right periventricular area (Figure 2a), suggesting a subacute ischemic phase. The leukoencephalopathy was found as mildly more extensive (Figure 2b,c). The Susceptibility weighted imaging (SWI) sequence also highlighted the presence of some microbleeds and left superficial hemosiderosis in the cerebral sulci at the brain convexity (Figure 2d). The autoimmune assessment showed a persistent mild elevated LAC confirmed by dRVV ratio (value 1.28).

## 2. Discussion

Partial 13q trisomy is most often reported to be associated to a variable neuroradiological cerebral picture including cerebral malformations as well as cortical dysplasia, holoprosencephaly, callosal dysgenesis, hippocampal and vermian hypoplasia; other types of cerebral radiological findings like ischemic or haemorrhagic brain lesions are less reported and are not considered as part of the clinical phenotype [4]. So far, Ribacoba et al. [4] reported the history of a 33-year-old female carrying a partial trisomy 13q22-qter who suddenly suffered left hemiplegia and generalised epilepsy related to an ischemic stroke. In our case, the patient presented with left hemiparesis associated to both ischemic and haemorrhagic stroke related to cerebral vasculitis.

Stroke is known to be a complex disease caused by a combination of multiple risk factors, including a strong genetic component [5,6]. Recent reports proposed that both ischemic and hemorrhagic stroke could share a common genetic basis regarding gene variants of the clotting system [6,7]. In our case, stroke was associated to hemorrhagic and ischemic events related to cerebral vasculitis in presence of hyperhomocysteinemia (HHCys) associated to heterozygotic MTHFR C6771 polymorphism. Both the World Health Organization (WHO) and the Health Ministry considered HHCys a strong contributor for cardiovascular disease as persistent HHCys values lead to the formation of atherosclerotic plaques, atherothrombotic events through endothelial dysfunction [8]. HHCys is also found to be more evident as responsible of coronary events respect to controls in autoimmune diseases including rheumatoid arthritis (RA) and systemic lupus erythematosus (LES) [8]. However, the predictability of the association between MTHFR C677T polymorphism and thrombophilia is currently denied by several reports [9].

We also investigate the function of different genes involved in the duplicated 13q22-q34 region and their possible role on both immunological functions and coagulation pathways. Some of these genes are involved in the coagulation pathway as the Z protein (*PROZ*) and the Growth arrest-specific gene 6 protein (*GAS6*) genes, known to play an important role in regulating blood coagulation and in the regulation of thrombotic responses as well as in cerebral haemorrhage, thrombosis and in the genetic susceptibility of systemic lupus erythematosus (SLE) [5,10,11,12,13,14,15]. In our patient cerebral vasculitis findings associated to LAC positivity could be related also to the involvement of the *TNFSF13B* (named also B lymphocyte stimulator, *BLyS*) gene that encodes a cytokine that belongs to the tumor necrosis factor (TNF) ligand family and it is reported to be significantly increased in the primary antiphospholipid syndrome (PAPS) and in LES, as confirmed by an animal model study [16,17,18,19].

The other neurological findings of our patient related to the presence of intellectual disability and epilepsy could be correlated to *STK24* (also called *MST3*) gene, that belongs to the MST kinases, playing an emerging role in modulation of excitatory synaptic transmission in epileptic hippocampal mice neurons, through the interactions with NMDAR subunits [20,21]. However, another gene that could play an important role is the *NALCN* gene, expressed in different regions of the brain and encoding voltage sodium and calcium channels that regulate the resting membrane potential and neurons excitability [22,23]. Finally, as reported in other studies, *GPC5* and *GPC6* genes are involved in limb formation and are responsible for postaxial polydactyly type A2 that was also present in our patient [24].

## 3. Conclusions

Thus, 13q22-q34 is a crucial region that could be associated to a late onset of ischemic and hemorrhagic stroke. This is the second clinical case affected by partial 13q trisomy reported in the literature that presented with stroke and hemiparesis. We found a genetic predisposition that could explain the link between the autoimmune and the coagulation system. Antiphospholipid antibodies could represent the key of interaction that should be checked even in genetic disorders presenting with stroke, as they could help to address a more specific anticoagulant therapy in order to prevent the evolution of the cerebrovascular disease. However, further studies are needed in order to confirm the role of the genes involved in the 13q22-q34 on immunological and neurological findings to better define genotype and phenotype correlation.

## Data Availability

Data sharing not applicable.
